# Arithmetic skills are associated with left fronto-temporal gray matter volume in 536 children and adolescents

**DOI:** 10.1038/s41539-023-00201-x

**Published:** 2023-12-08

**Authors:** Nurit Viesel-Nordmeyer, Jérôme Prado

**Affiliations:** 1 Lyon Neuroscience Research Center (CRNL), INSERM U1028-CNRS UMR5292, University of Lyon, 69500 Bron, France; 2 Department of Rehabilitation Sciences, TU Dortmund University, Dortmund, Allemagne; 3 Laboratoire de Psychologie Cognitive, Aix-Marseille University & CNRS, Marseille, France

**Keywords:** Cognitive neuroscience, Human behaviour

## Abstract

There are large individual differences in arithmetic skills. Although a number of brain-wide association studies have attempted to identify the neural correlates of these individual differences, studies have focused on relatively small sample sizes and have yielded inconsistent results. In the current voxel-based morphometry study, we merged six structural imaging datasets of children and adolescents (from 7.5 to 15 years) whose levels of arithmetic skills were assessed, leading to a combined sample of *n* = 536. Controlling for individual differences in age, gender, as well as language, and intelligence, we found a unique positive relation between arithmetic skill and gray matter volume in the left inferior frontal gyrus (IFG) and middle temporal gyrus (MTG). Our results suggest that individual differences in arithmetic skills are associated with structural differences in left fronto-temporal areas, rather than in regions of the parietal cortex and hippocampus that are often associated with arithmetic processing.

## Introduction

Although numeracy is crucial for education and social participation in our modern society^1^, studies regularly point to large disparities in math skills as early as in elementary school^1,2^. This suggests that these disparities can be traced back to differences in foundational numerical skills, and notably to differences in the ease with which individuals can solve simple arithmetic problems^3^. Therefore, it is important to improve our understanding of the neuro-cognitive mechanisms underlying individual differences in arithmetic skills to inform instruction and assessment.

Functional imaging studies suggest that a number of brain areas are involved in arithmetic processing^4^. These notably include regions of the parietal cortex, such as the intraparietal sulcus and left angular gyrus. Because the intraparietal sulcus (IPS) has long been thought to support the representation of symbolic and non-symbolic numerical quantity^5^, it has been argued that it may be involved in the manipulation of numbers during mental calculation^4–6^. In contrast, the left angular gyrus (AG) is particularly activated when participants solve arithmetic problems that are reported to be retrieved from memory^7^, which suggests a specific role for this region in the retrieval of solutions from long-term memory^7^.

However, studies have also found that arithmetic processing is supported by a number of other brain regions. For example, it has been suggested that, in addition to the left AG, the left middle temporal gyrus (MTG) might critically support the retrieval of solutions from memory given its role in phonological processing^8^. This idea is supported by several studies that have demonstrated that the left MTG is involved when participants are presented with problems that have been learned by rote in school, such as single-digit multiplication^9–11^. Studies also implicate regions of the left inferior frontal gyrus (IFG), particularly when calculation becomes more demanding and taxes verbal working memory^9,10,12^. Finally, studies in children also suggest that the hippocampus might play a role in the encoding and retrieval of associations between arithmetic problems and answers, particularly during the early stages of arithmetic learning^12,13^.

Although the studies above have undoubtedly helped identify the overall brain regions subserving arithmetic processing across individuals, they do not necessarily inform about the neural correlates of *individual differences* in arithmetic skills. Can such individual differences be linked to individual differences in brain function and structure? Some functional neuroimaging studies (using functional magnetic resonance imaging, infrared spectroscopy, or electroencephalography) have attempted to answer that question by comparing patterns of brain activity during math tasks in individuals with lower versus higher math skills. These studies, however, have led to relatively heterogeneous results. In young adults, for example, reduced activation in both the left IFG and temporal cortex during mental arithmetic has been observed in lower-skilled individuals^14,15^. Other studies have found a reduced modulation of parietal and prefrontal responses with increasing arithmetic complexity in children with math learning difficulty as compared to their typically developed peers^16,17^. More generally, studies are inconsistent regarding the location and direction of differences observed between lower and higher-skilled children^16–18^.

A smaller number of studies have also focused on the structural correlates of individual differences in arithmetic skill^4,19^. Most studies comparing individuals with lower versus higher math skills have found reduced gray matter volume (GMV) in math-impaired children in the parietal lobe as well as in the bilateral inferior or middle frontal gyrus^20–22^. Studies that examined brain-behavior correlations across entire samples have found relations between arithmetic skill and GMV in the parietal lobe^23,24^, but also in the fusiform gyrus^19^, hippocampus^25^, and ventrotemporal occipital cortex^23^. Recently, Suárez-Pellicioni and colleagues^24^ found a positive relation between GMV of the left MTG and multiplication skills in 10–12-year-olds. Overall, then, neuroimaging studies have identified a number of structural correlates of arithmetic skills encompassing parietal, frontal, temporal, but also hippocampal regions^25^.

A major shortcoming of these previous neuroimaging studies, however, is that they were conducted with relatively small numbers of participants. For example, in their review of the literature, Peters and de Smedt^4^ indicate that sample sizes of structural brain-wide association studies (BWAS) investigating correlates of arithmetic skills ranged from *n* = 11 to *n* = 59, with an average of *n* = 24. As emphasized in a growing number of reports^26,27^, BWAS with such relatively small sample sizes may be characterized by effect sizes that are inflated and difficult to replicate because studies are underpowered to detect functional or structural brain-behavior associations. A recent study investigating associations between GMV and math skills in a relatively large sample of children (*n* = 224) already suggests that this might be the case. Indeed, after adjusting for total brain volume, the authors found no concurrent associations between GMV and math skills at age 7 and only a unique association with GMV in the left superior temporal cortex at age 13^28^. Yet, most children included in that study were born preterm, and even that sample size remains modest for a BWAS^26,27^.

To address the power and reliability issue of BWAS of arithmetic skills, we gathered here data from six different datasets^29–34^, leading to a combined sample size of *n* = 536. Each dataset included structural brain imaging of children or adolescents whose levels of arithmetic skills were measured outside of the scanner (as part of a comprehensive behavioral testing session). We then used voxel-based morphometry (VBM) to assess the relation between levels of arithmetic skills and GMV in several brain regions that have been identified as supporting mental arithmetic in previous studies, while controlling for a number of other languages (vocabulary and reading skills) and cognitive (IQ) skills that are known to play a role in arithmetic learning^35^. To our knowledge, our study is the largest BWAS of arithmetic skills to date.

## Results

### Samples

Demographic information about the six datasets included in the present study is shown in Table 1. These datasets are hereafter referred to as set #1^29^, set #2^30^, set #3^31^, set #4^32^, set #5^33^ and set #6^34^. Participants from set #1 and set #2 come from the Lyon area in France while participants from set #3 to set #6 come from the greater Chicago metropolitan area in the United States (US). Note that different models of scanners were used for data acquisition at these two sites (see “Methods”). Our final sample includes 536 children and adolescents from age 7.5 to age 15 (mean = 10.58, *SD* = 1.60).

**Table 1:** Demographic information.

Dataset	*n*	Age		Sex (female)	Native language	Scanning location
		Mean (*SD)*	Range			
Set #1	53	8.47 (35)	8.01–9.24	30.2 %	French	CERMEP, Lyon, France
Set #2	42	11.05 (1.25)	8.47–11.05	54.8 %	French	CERMEP, Lyon, France
Set #3	132	11.26 (1.46)	8.36–15.00	53.0 %	English	CAMRI, Chicago Illinois, US
Set #4	185	10.48 (1.62)	7.50–14.38	48.1 %	English	CAMRI, Chicago Illinois, US
Set #5	56	11.20 (1.64)	8.47–15.00	57.1 %	English	CAMRI, Chicago Illinois, US
Set #6	68	10.34 (.94)	8.59–11.96	5.9 %	English	CAMRI, Chicago Illinois, US
Total	536	10.58 (1.60)	7.50–15.00	43.7 %	NA	NA

*CAMRI* Northwestern University Center for Advanced Magnetic Resonance Imaging, *CERMEP* Center d’Exploration et de Recherche Multimodale et Pluridisciplinaire.

### Behavioral results

Results of descriptive analyses for all behavioral data, including control variables of sex, scanning site, attention deficit hyperactivity disorder (ADHD) status, and age can be found in Table 2. Because different instruments were used for measuring children’s skills in the six datasets (see “Methods”), raw scores were *z*-transformed within each dataset and are shown in *z*-standardized form for the full sample (*n* = 536) in the table. Separate information for the six datasets with the original mean and standard deviation of the measurements are shown in the supplemental file (see Tables S1–6).

**Table 2:** Descriptive statistics and correlations for behavioral variables in the full sample (*n* = 536).

Variables	Cat. 1	%	Cat. 2	%	(1)	(2)	(3)	(4)	(5)	(6)	(7)	(8)
(1) Sex	Male	56.3	Female	43.7								
(2) Scanning site	F	17.7	US	82.3	0.02							
(3) ADHD	no	78.4	yes	21.6	–0.18**	0.24**						
	Mean	*SD*	Min	Max								
(4) Age	10.58	1.60	7.50	15.00	–0.05	0.28**	0.03					
(5) Verbal IQ	0.00	1.00	–3.45	2.37	0.03	0.00	–0.10*	−0.07				
(6) Non-verbal IQ	0.00	1.00	–3.46	2.04	0.01	0.00	–0.10*	–0.12**	0.41**			
(7) Arithmetic	0.00	1.00	–3.01	2.49	–0.01	0.00	–0.18**	–0.05	0.43**	0.46**		
(8) Vocabulary	0.00	1.00	–2.49	2.65	0.02	0.00	–0.08	–0.11**	0.68**	0.44**	0.44**	
(9) Reading	0.00	1.00	–3.70	3.22	0.12*	0.00	–0.14**	–0.09*	0.35**	0.31**	0.40**	0.40**

Pearson correlation, *p ≤ 0.05, **p ≤ 0.01. Scores of IQ verbal, IQ non-verbal, vocabulary, reading, and arithmetic are *z*-standardized because different measurements were used in the six datasets (see Table S1). Cat. 1 = Category 1, Cat. 2 = Category 2. F = France, US = United States. Significant (*p* < 0.05) correlations are in bold.

Correlations between measures of verbal and non-verbal IQ, vocabulary, reading, and arithmetic skills were medium to large across the whole sample. Scanner site was only associated with age and ADHD status, mainly because (1) sets #3 to #6 were collected on children who were older than in sets #1 and #2 and (2) only sets #3 to #6 included children diagnosed with ADHD. Sex was only weakly correlated to reading skills across the whole sample. In line with previous research^36^, there was also a small negative correlation between ADHD status and all skills (with the exception of vocabulary).

### VBM results

**Fig. 1:**
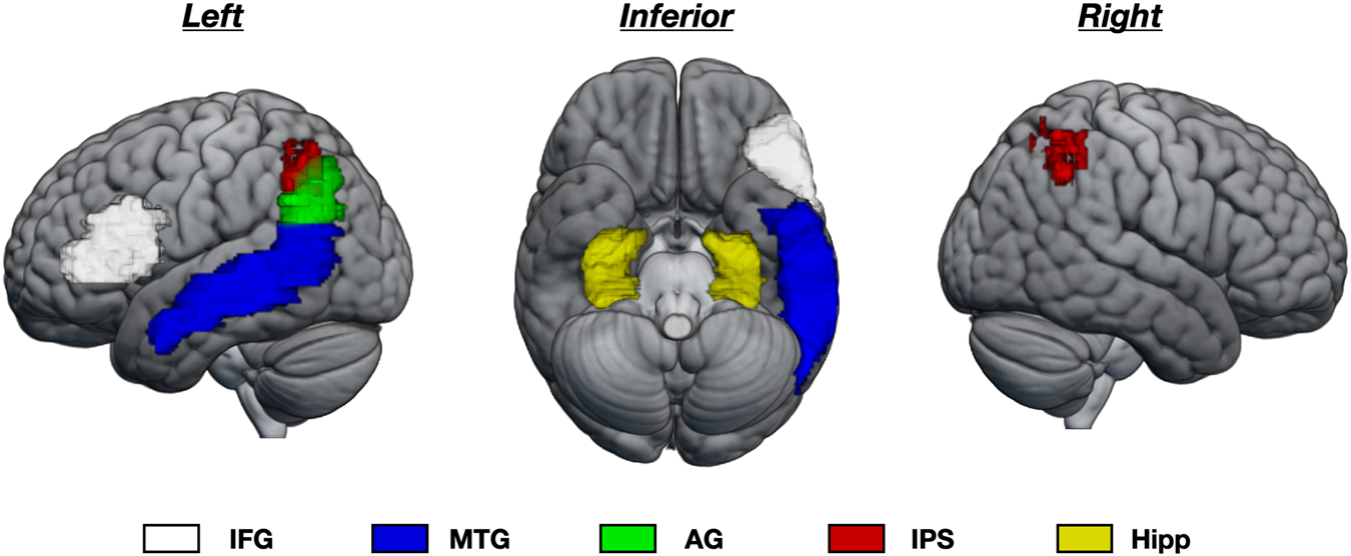
ROI locations. Location of ROIs displayed on a rendering of the MNI-normalized brain.

For each participant and each dataset, average GMV was extracted from seven regions of interest (ROIs) that have been found associated with mental arithmetic in previous studies (see “Introduction”). All ROIs were defined anatomically to avoid circularity in analyses^37^ (see “Methods”). These ROIs were the bilateral IPS, the left AG, the left MTG, the left IFG, and the bilateral hippocampus (see Fig. 1). For each ROI, GMV was entered in a linear mixed-effect model to analyze its relation with arithmetic skill, while taking into account the nested structure of the data. Fixed-effects covariates were total intracranial volume (TIV), age, sex, and ADHD. Because sets differed with respect to the scanning site (France versus US) and arithmetic test (WJ-III versus CMAT), these were considered random effects. Across all mixed-model analyses, a relation between GMV and arithmetic score was only observed in the left IFG (Table 3) and left MTG (Table 4). Results from other ROIs are shown in the supplemental file (see Tables S8–12) (note that an exploratory whole-brain analysis confirming relations between GMV and arithmetic score in the left IFG and left MTG is presented in the supplementary information, see Fig. S1).

**Table 3:** Mixed-model analysis of GMV in the left IFG without covariates of language and cognitive measures.

Fixed effects	Estimated coefficient	*SE*	Lower	Upper	*df*	*t*	*p*
(Intercept)	0.410	0.00	0.403	0.417	1.08	114.49	0.00
Arithmetic	0.004	0.00	0.001	0.006	528.96	**2.79**	**0.01**
ADHD (1–0)	–0.003	0.00	–0.010	0.004	503.77	–0.80	0.43
Sex (2–1)	0.001	0.00	–0.006	0.008	528.22	0.31	0.76
Age	–0.003	8.05e-4	–0.005	–0.001	416.32	**–3.71**	**<** **0.001**
TIV	2.67e-4	1.04e-5	2.47e-4	2.88e-4	515.33	**25.61**	**<** **0.001**
Sex*ADHD	–0.002	0.01	–0.015	0.011	528.32	–0.28	0.78
*Random effects*	*Variance*	*SD*	*ICC*				
Arithmetic Test (Intercept)	1.19e-19	3.45e-10	1.44e-16				
Scanning Site (Intercept)	1.72e-5	0.00	0.02				
Residual	8.26e-4	0.03					

Random effects are correlated. AIC = –2268.02; BIC = –2141.83; *R*-squared marginal = 0.64; *R*-squared conditional = 0.64. Significant (*p* < 0.05) fixed effects are in bold.

**Table 4:** Mixed-model analysis of GMV in the left MTG without covariates of language and cognitive measures.

Fixed effects	Estimated coefficient	*SE*	Lower	Upper	*df*	*t*	*p*
(Intercept)	0.466	0.00	0.460	0.471	1.05	176.44	0.00
Arithmetic	0.004	0.00	0.002	0.006	528.69	**3.54**	< **0.001**
ADHD (1–0)	0.003	0.00	–0.002	0.009	484.20	1.20	0.23
Sex (2–1)	0.006	0.00	3.48e-4	0.011	528.28	1.85	0.07
Age	–0.003	6.71e-4	–0.005	–0.002	357.50	**–4.88**	**<** **0.001**
TIV	3.12e-4	8.72e-6	2.95e-4	3.29e-4	503.57	**35.80**	**<** **0.001**
Sex*ADHD	–0.005	0.01	–0.006	0.016	528.40	0.96	0.34
*Random effects*	*Variance*	*SD*	*ICC*				
Arithmetic Test (Intercept)	1.19e-19	3.45e-10	1.44e-16				
Scanning Site (Intercept)	1.72e-5	0.00	0.02				
Residual	8.26e-4	0.03					

Random effects are correlated. AIC = –2460.43; BIC = –2331.15; *R*-squared marginal = 0.77; *R*-squared conditional = 0.77. Significant (*p* < 0.05) fixed effects are in bold.

Frequentist statistics, however, cannot provide evidence for a null hypothesis. In other words, a nonsignificant relation between GMV and arithmetic score in a given ROI does not mean that a relation does not exist. Therefore, we used Bayesian mixed-effect models to estimate the strength of evidence (i.e., the Bayes factor, BF) for the null hypothesis of no relation between GMV and arithmetic score (H0) versus the alternate hypothesis of a relation (H1) in each ROI. Evidence for a lack of relation between GMV and arithmetic score was strong in the left AG (BF_01_ = 11.58) and hippocampus (left: BF_01_ = 10.38, right: BF_10_ = 12.43), substantial in the left IPS (BF_01_ = 7.10), and anecdotal in the right IPS (BF_01_ = 2.82). In contrast, there was substantial evidence for a relation between GMV and arithmetic score in the left IFG (BF_10_ = 4.95), and very strong evidence for that relation in the left MTG (BF_10_ = 37.61). Therefore, not only do the results show that arithmetic score relates to GMV in the left IFG and left MTG, there is also evidence that it does not relate to GMV in the other ROIs.

To further evaluate the specificity of the relation between arithmetic skill and GMV in the left IFG and left MTG, we included other language and cognitive measures as covariates in the frequentist mixed-model analyses. Results indicated that the relation between arithmetic skill and GMV remained significant when adding as covariate reading skill (left IFG: *β* = 0.004, SE = 0.001, *t* = 3.169, *p* = 0.002; left MTG: *β* = 0.004, SE = 0.001, *t* = 3.604, *p* < 0.001), vocabulary skill (left IFG: *β* = 0.004, SE = 0.001, *t* = 2.459, *p* = 0.014; left MTG: *β* = 0.004, SE = 0.001, *t* = 2.741, *p* = 0.006), non-verbal IQ (left IFG: *β* = 0.003, SE = 0.002, *t* = 2.258, *p* = 0.024; left MTG: *β* = 0.003, SE = 0.001, *t* = 2.688, *p* = 0.007), and verbal IQ (left IFG: *β* = 0.004, SE = 0.002, *t* = 2.304, *p* = 0.022; left MTG: *β* = 0.003, SE = 0.001, *t* = 2.569, *p* = 0.010). The relation between arithmetic skill and GMV also remained significant when all four covariate measures were included in the model (see Table 5 for the left IFG and Table 6 for the left MTG). Therefore, there was a relation between arithmetic skill and GMV in these ROIs over and above individual differences in verbal and non-verbal skills.

**Table 5:** Mixed-model analysis of GMV in the left IFG with covariates of language and cognitive measures.

Fixed effects	Estimated coefficient	*SE*	Lower	Upper	*df*	*t*	*p*
(Intercept)	0.409	0.00	0.402	0.416	1.08	114.30	0.00
Arithmetic	0.004	0.00	5.32e-4	0.007	524.14	**2.30**	**0.02**
Reading	–0.002	0.00	–0.006	4.63e-5	524.54	–1.93	0.05
Vocabulary	0.003	0.00	–6.36e-4	0.006	524.22	1.61	0.11
Comparison	–9.02e-4	0.00	–0.004	0.003	524.00	–0.52	0.60
Matrix	5.17e-4	0.00	–0.002	0.003	524.80	0.34	0.73
ADHD (1–0)	–0.003	0.00	–0.010	0.003	500.10	–0.97	0.33
Sex (2–1)	0.001	0.00	–0.006	0.009	524.45	0.37	0.71
Age	–0.003	8.16e-4	–0.005	–0.001	397.92	**–3.59**	**<** **0.001**
TIV	2.67e-4	1.09e-5	2.46e-4	2.88e-4	502.25	**24.58**	**<** **0.001**
Sex*ADHD	–0.003	0.01	–0.016	0.010	524.27	–0.41	0.69
*Random effects*	*Variance*	*SD*	*ICC*				
Arithmetic Test (Intercept)	0.00	0.00	0.00				
Scanning Site (Intercept)	1.73e-5	0.00	0.02				
Residual	8.24e-4	0.03					

Random effects are correlated. AIC = –2265.85; BIC = –2077.90; *R*-squared marginal = 0.64; *R*-squared conditional = 0.64. Significant (*p* < 0.05) fixed effects are in bold.

**Table 6:** Mixed-model analysis of GMV in the left MTG with covariates of language and cognitive measures.

Fixed effects	Estimated coefficient	*SE*	Lower	Upper	*df*	*t*	*p*
(Intercept)	0.466	0.00	0.460	0.471	1.03	179.29	0.00
Arithmetic	0.003	0.00	7.88e-4	0.01	524.18	**2.57**	**0.01**
Reading	–0.002	0.00	–0.004	7.08e-4	524.68	–1.38	0.17
Vocabulary	0.002	0.00	–9.15e-4	0.005	524.29	1.35	0.18
Comparison	0.001	0.00	–0.002	0.004	524.00	0.77	0.44
Matrix	–4.17e-4	0.00	–0.003	0.002	524.94	–0.33	0.74
ADHD (1–0)	0.003	0.00	–0.003	0.009	477.39	1.10	0.27
Sex (2–1)	0.006	0.00	–3.68e-4	0.012	524.57	1.84	0.07
Age	–0.003	6.79e-4	–0.005	–0.002	326.18	**–4.73**	**<** **0.001**
TIV	3.11e-4	9.07e-6	2.93e-4	3.29e-4	481.17	**34.30**	**<** **0.001**
Sex*ADHD	0.005	0.01	–0.006	0.016	524.36	0.93	0.35
*Random effects*	*Variance*	*SD*	*ICC*				
Arithmetic Test (Intercept)	0.00	0.00	0.00				
Scanning Site (Intercept)	7.85e-6	0.00	0.01				
Residual	5.76e-4	0.02					

Random effects are correlated. AIC = –2458.45; BIC = –2265.90; *R*-squared marginal = 0.77; *R*-squared conditional = 0.77. Significant (*p* < 0.05) fixed effects are in bold.

## Discussion

Previous studies have shown relations between arithmetic skills (or math skills more broadly) and neuroanatomy in a variety of brain areas, including regions of the parietal, frontal, occipital, and temporal cortex, as well as the hippocampus^19–25^. Controlling for individual differences in a range of language and cognitive skills and studying the largest sample of participants to date, we found here that individual differences in arithmetic skills were related to individual differences in GMV of the left fronto-temporal cortex rather than areas of the parietal cortex or hippocampus that have also been implicated in arithmetic studies^4^.

With the exception of Ranpura et al.^21^ and Rotzer et al.^22^ who found a decrease in GMV of the left IFG in children with math learning difficulty, most previous studies have not identified the left IFG as a neuroanatomical substrate of arithmetic skills. However, this region has been identified in several functional neuroimaging studies. For example, Yang and colleagues^38^ found greater activity for subtraction compared to addition in a number of regions on the left hemisphere, including the IFG in adults. Studies have also indicated increased activity in the left IFG with problem complexity^15,38–40^. Chang et al.^41^ found enhanced activity in the left IFG during arithmetic processing in children, while de Smedt et al.^12^ found greater activity in this region for large versus small arithmetic problems. Evans et al.^42^ also found greater left IFG activity for single-digit addition than an active control condition in the left IFG in a sample of adults and children.

In contrast to the left IFG, a positive relation between arithmetic skills and GMV has previously been reported in the MTG. For example, Suárez-Pellicioni and colleagues^24^ showed a positive association between GMV and multiplication skills in the MTG. McCaskey et al.^20^ also demonstrated reduced GMV in the MTG in children with dyscalculia compared to their typically developed peers. Therefore, our study replicates these findings with a larger sample of participants. The involvement of the left MTG in arithmetic processing is further suggested by several functional neuroimaging studies. For example, Prado and colleagues^9^ found enhanced activity in the left MTG when adults solve single-digit multiplication problems, while Prado and colleagues^10^ found age-related increases of activity in that region in children solving the same task. Activity in the left temporal cortex has also been shown to increase with arithmetic fact fluency^15^. In a longitudinal study, Suárez-Pellicioni and colleagues^43^ demonstrated that age-related decreases of connectivity between the left MTG and the left IFG support efficient learning of multiplication facts.

As is the case in all BWAS investigating structural brain-behavioral correlations, we can only speculate about the specific cognitive processes supported by the left IFG and left MTG during mental arithmetic. For instance, it has been proposed that the left MTG might support the association between arithmetic facts and their answers through their phonological codes^8^, consistent with the role of this region in phonological processing^44^. This would be broadly consistent with the idea that arithmetic learning is characterized by a shift from procedural (e.g., counting) to verbal retrieval, such that operands and answers of at least some single-digit problems would become associated in memory through their phonological codes^45,46^.

Note that associations between operands and answers within a network of facts are likely to lead to verbal interferences, notably as the size of the problem increases^45^. Suppression of verbal interferences has been attributed to the left IFG and some have proposed that this might explain the involvement of this region in mental arithmetic^47^. However, others have also argued that the left IFG might support mental arithmetic because of its role in the sequential processing of linguistic stimuli. For instance, Nakai and colleagues^48,49^ found shared processing of arithmetic and linguistic syntax in the left IFG. Evans et al.^42^ found enhanced activity in the same region of the left IFG in single-digit addition and word reading tasks, suggesting that the region supports processes common to arithmetic and reading. An earlier study also found shared activity between verbal working memory and digit processing in the left IFG^50^. Again, note that the above interpretations largely rely on reverse inferences and need to be interpreted with caution^51^. For instance, Ashkenazi and colleagues^52^ found in the left IFG a positive correlation between activity associated with complex addition and block recall, which is a measure of visuo-spatial rather than verbal working memory. Therefore, it is also possible that the left IFG also contributes to mental arithmetic through its role in visual attention^53^. Functional neuroimaging studies are the best positioned to shed light on the role of the left IFG and left MTG in mental arithmetic.

To our knowledge, our study is the largest structural BWAS of arithmetic skills to date. However, it is important to acknowledge a number of limitations. First, arithmetic skills were defined based on the calculation subtest of the WJI-III^54^ for four of the datasets. Because the test merges different types of arithmetic operations (addition, subtraction, multiplication, division), it is not possible to evaluate whether the link between GMV and arithmetic skills changes with the type of operation. Second, we assessed individual differences in brain structure using VBM. It is possible that associations between arithmetic skills and other brain regions might be found with other types of measures (e.g., deformation-based morphometry^25^; surface-based analyses^21^). In fact, we performed an exploratory analysis of the relation between cortical thickness (CT) and arithmetic skills using the CAT12 toolbox on the same sample of participants (including the same covariates as in our main VBM analysis). This analysis did not reveal any significant relation across the whole brain (see “Methods”). Unlike CT, VBM captures a mixture of measures of gray matter, including cortical surface area and cortical folding in addition to cortical thickness. Therefore, it is possible that the relation between arithmetic skills and left IFG structure may specifically relate to cortical folding or cortical surface area, though this needs to be investigated in future studies. Finally, although our overall sample size of more than 500 participants is a significant improvement in the literature investigating relations between brain structure and arithmetic skills, it remains limited and does not allow us to perform reliable additional analyses with subgroups of participants (e.g., split by age)^26,27^. Future well-powered studies with more specific age groups are needed to investigate how the relation between brain structure and arithmetic skills changes with age.

In sum, our results highlight brain-wide associations between arithmetic skills and GMV of the left IFG and left MTG in the largest sample of children and adolescents to date. To some extent, these results conflict with previous BWAS of arithmetic skills that have often identified the parietal cortex as a structural correlate of individual differences in arithmetic skills. Here not only did we not find any relation between arithmetic skill and GMV in the parietal cortex, Bayesian analyses indicated evidence for a lack of relation. More generally, our results emphasize the need to study associations between math skills and brain structure using large sample sizes, in line with current recommendations in the field^26^.

## Methods

### Sample

Across all six datasets (see Table 1), exclusion criteria included hearing deficit, magnetic resonance imaging (MRI) contraindication, history of neurological and psychiatric disorders, prematurity less than 36 weeks, and medication affecting central nervous system processing. Participants with a diagnosis of ADHD were only excluded from set #1 and set #2, but not from set #3 to set #6 (see Tables S2 to S7 for proportion). Note that, although set #6 included a relatively high proportion of children and adolescents with a clinical diagnosis of ADHD, participants were instructed to not take stimulant medication for at least 24 h prior to the testing sessions^34^. Also note that although set #4 and set #6 originally included 188^32^ and 79^34^ participants, three participants from set #4 and 11 participants from set #6 had to be excluded from the present analyses because of missing data of interest. One participant from set #1 also had to be excluded because of image artifacts (see below). Informed written consent for study participation was provided by parents and participation was consented to by children. Data collection for set #1 and set #2 was approved by a French national ethics committee (CPP Lyon Sud-Est II), while data collection for set #3 to set #6 was approved by the Institutional Review Board at Northwestern University in the US.

### Behavioral assessment

Children’s skills in arithmetic and in a range of other cognitive and academic skills (vocabulary, reading, verbal and non-verbal IQ) were assessed in all six datasets. The instruments of interest are indicated in Table S1.

In all datasets, arithmetic skills were assessed using the *calculation subtest* of the *Woodcock-Johnson III Tests of Achievement* (WJ-III)^54^ or the *Basic Calculations Composite* of the *Comprehensive Mathematical Abilities Test* (CMAT)^55^. Both tests are untimed paper- and pencil tests in which children solve increasingly difficult arithmetic problems from the four operations (addition, subtraction, multiplication, division). An aggregate score including the four operations is used in the present study. Each correct answer is scored 1 and each incorrect answer is scored 0.

Vocabulary skills were assessed in each dataset using *vocabulary subtests* from either the *Nouvelle Echelle Métrique de l’Intelligence-2* (NEMI-2)^56^ or the *Wechsler Abbreviated Scale of Intelligence* (WASI)^57^. In both subtests, children have to orally define words that are presented visually and orally. Each correct answer is scored 1 and each incorrect answer is scored 0.

Reading skills were assessed in all datasets using the *indice de precision* (CM) of the *Alouette reading test*^58^ (set #1 and set #2) or the *Sight Word Efficiency Subtest* (SWE) of the *Test of Word Reading Efficiency* (TOWRE)^59^ (sets #3 to #6). In the Alouette test, children read a nonsensical text in 2 min. The CM is calculated by dividing the number of words correctly read by the number of words in the text (multiplied by 100). In the TOWRE, children have to pronounce real words that are printed on paper within 45 s. The level of difficulty increases from single syllables to multiple syllables. A reading score is calculated based on reading accuracy and number of words read.

Verbal IQ was measured in all datasets either using the *comparison subtest* of the NEMI-2^56^ or the *similarities subtest* of the WASI^57^. In these subtests, participants have to find the common characteristics between different verbal terms. Each correct answer is scored 1 and each incorrect answer is scored 0.

Non-verbal IQ was measured in all datasets using the *Matrix subtests* of the NEMI-2^56^ and of the WASI^57^. In both tests, an incomplete matrix of shapes was shown to the children, who had to select the response option that completes the matrix. Each correct answer is scored 1 and each incorrect answer is scored 0.

### MRI data acquisition

In each of the six datasets, high-resolution anatomical scans were collected during the MRI session. In set #1 and set #2, brain imaging data were acquired using a 64-channel head coil and a Siemens 3 T Prisma Scanner (Siemens Healthcare, Erlangen, Germany). Parameters of the anatomical scan for set #1 and #2 were as follow: TR = 3500 ms, TE = 2.24 ms, flip angle = 8°, matrix size = 256 × 256, slice thickness = 0.90 mm, number of slices = 192, voxel size resolution = 0.875 mm isotropic. In sets #3 to #6, brain imaging data were acquired using either a 16-channel or a 32-channel head coil and a Siemens 3 T Trio-Tim Scanner (Siemens Healthcare, Erlangen, Germany). Parameters of the anatomical scan for sets #3 to #6 were as follows: TR = 2300 ms, TE = 3.36 ms, flip angle = 9 °, matrix size = 256 × 256, slice thickness = 1 mm, number of slices = 160, voxel size resolution = 1 mm isotropic.

### VBM analyses

Structural images were analyzed using the Computational Anatomy Toolbox (Cat 12)^60^ within the Statistical Parametric Mapping Software Package (SPM 12)^61^. Preprocessing of all images included the following steps. First, images were segmented into GM, WM, and cerebrospinal fluid (CSF) images using Tissue Probability Maps provided in the CAT12 toolbox^62^. Second, images were spatially normalized using DARTEL registration^63^ with an MNI template also provided by the CAT12 toolbox^60^. At that point, normalized and segmented images were systemically checked for artifacts or orientation issues (one participant from set #1 had to be excluded at that stage). Third, to account for brain differences in size and volume, TIV was estimated. Data homogeneity was also checked for possible outliers (no participant was excluded). Finally, GM images were spatially normalized and smoothed with an 8 mm^3^ Gaussian Kernel.

Based on previous functional neuroimaging studies on arithmetic processing (see **Introduction**), we focused on seven anatomically defined regions of interest (ROIs). These included five ROIs from the automated anatomical atlas 3 (AAL3)^64^: the left AG, the left MTG, the left IFG (including the left opercular and triangular part), and the bilateral hippocampus. We also used the Anatomy Toolbox (Version 2.2^65^) to define ROIs in the left and right IPS. Following several of our previous studies^30^, these IPS ROIs consisted of voxels with at least a 50% probability of belonging to one of the IPS subdivisions (hIP1, hIP2, and hIP3), as defined in the Anatomy Toolbox.

In each ROI, differences in GMV between participants were assessed by evaluating differences in mean voxel intensity from GM images. Specifically, for each participant, we extracted the mean voxel intensity within an ROI by averaging the values across all voxels within that ROI. Note that mean voxel intensities are not absolute values of GMV (which typically take into account the number of voxels in ROI and volume per voxel). However, individual differences in mean voxel intensity are a proxy for the relevant dimension in the current study, i.e., individual differences in GMV (number of voxels in ROI and volume per voxel being held constant across participants).

Mean intensity from each ROI was entered as the dependent variable in several linear mixed-model analyses to evaluate the significance of the relation between GMV and arithmetic skill, taking into consideration the nested structure of the data. Fixed effects systematically included TIV, ADHD status, age, and sex. Additional models included vocabulary, reading, verbal and non-verbal IQ as covariates to evaluate whether these affected a potential relation between arithmetic skill and GMV. In all analyses, random effects included type of arithmetic test (WJI-III, CMAT) and scanning site (France, US). To preserve model parsimony^66^ in all models and for each ROI, we checked the improvement of model fit by including the slopes of the relation between GMV and arithmetic skill across arithmetic tests and scanning sites in addition to intercepts across sites. In all cases, more complex models including random intercepts and random slopes led to improvements in goodness of fit that were inferior to 5 % compared to models that only included random intercepts, which speaks against using the most complex models with random slopes^67^. This was also confirmed by likelihood-ratio tests^66^. Therefore, we only allowed the intercepts to vary across sites and arithmetic tests in all models. All analyses were conducted using Jamovi version 2.3.24^68^ including the jmv^69^ and GAMLj modules^70^.

Furthermore, to quantify the strength of evidence for a lack of relation between arithmetic score and GMV in each ROI, we used the BayesFactor package^71^ in R^72^ to compute mixed-model Bayesian analyses with mean voxel intensity as dependent variable, arithmetic score, TIV, ADHD status, age, and sex as fixed effects, and both arithmetic test and scanning site as random effects. Default priors as well as random intercepts were used. The BF associated with the relation between GMV and arithmetic skill was estimated by comparing mixed-effect models differing only in the presence or absence of the arithmetic score covariate. A BF < 3 was considered anecdotal evidence, a 3 < BF < 10 was considered substantial evidence, a 10 < BF < 30 was considered strong evidence, a 30 < BF < 100 was considered very strong evidence, and a BF > 100 was considered extreme evidence that our data are more likely under the alternate than the null hypothesis (i.e., BF_10_) or under the null hypothesis than the alternate hypothesis (i.e., BF_01_).

Finally, the relation between VBM and the arithmetic score was also analyzed using an exploratory linear regression model across the whole brain. Note that this analysis was exploratory because the whole-brain regression did not take into account the nested structure of the data. The variable of interest was arithmetic score and covariates were measures of TIV, ADHD status, age, sex, and scanning site. Clusters were considered significant if they survived a voxelwise Family-Wise Error rate corrected threshold of *p* < 0.05 (with a minimum cluster size of 0.30 cc), either across the whole brain or within an anatomical mask representing the union of all anatomically defined ROIs (see above) used in the main analyses (i.e., small volume correction). Results are shown in Supplementary Fig. 1.

### Exploratory cortical thickness (CT) analyses

In another set of exploratory analyses, we estimated CT^73^ using the surface-based morphometry (SBM) processing pipeline in the CAT12 toolbox^60^. Cortical thickness is a measure of the width of gray matter, calculated as the distance between the white and gray cortical surfaces. We used the default processing pipeline, which included five steps. First, cortical thickness and central surface for the left and right hemispheres were estimated using a projection-based thickness (PBT) method^74^, which also includes partial volume correction, sulcal blurring, and sulcal asymmetries without sulcus reconstruction. Second, topological correction was performed using a method based on spherical harmonics^75^. Third, to enable inter-participant comparison, an algorithm for spherical mapping of the cortical surface was used. Fourth, an adapted volume-based diffeomorphic DARTEL algorithm was applied to the surface for spherical registration^76^. The cortical thickness data were finally smoothed with a 15 mm full-width half-maximum Gaussian kernel. CT data were analyzed using linear regression models across the whole sample of participants. This analysis was also exploratory because it did not take into account the nested structure of the data. The variable of interest was arithmetic score and covariates were measures of TIV, ADHD status, age, sex, scanning site, as well as measures of language and IQ. This analysis did not show any relation between arithmetic score and CT (voxel-level threshold of *p* ≤ 0.001, cluster-level threshold of *p* ≤ 0.05, family-wise error corrected for multiple comparisons).

### Reporting summary

Further information on research design is available in the Nature Research Reporting Summary linked to this article.

### Supplementary information

Supplementary

Reporting Summary

## Data Availability

Raw MRI data are available on Open Neuro for sets #3 (https://openneuro.org/datasets/ds001486/versions/1.3.1), #4 (https://openneuro.org/datasets/ds002424/versions/1.2.0), #5 (https://openneuro.org/datasets/ds001894/versions/1.4.1), and #6 (https://openneuro.org/datasets/ds002886/versions/1.1.0). Individual behavioral and ROI data for all datasets are available from *Zenodo*, as well as ROI masks shown in Fig. 1 (10.5281/zenodo.7866555).
