# Supplementary material for: Arithmetic skills are associated with left fronto-temporal gray matter volume in 536 children and adolescents
Source: NPJ Sci Learn. 2023 Dec 8;8:56. doi: 10.1038/s41539-023-00201-x (PMC10709444; doi:10.1038/s41539-023-00201-x)
Supplement: Supplementary file 1 — Supplementary [file 41539_2023_201_MOESM1_ESM.docx]

**Supplementary**

**Supplementary Table 1.**

Assessment instruments for the six data sets

| Data set | IQ | | Language | | Arithmetic |
| --- | --- | --- | --- | --- | --- |
|  | verbal | nonverbal | vocabulary | reading |  |
| Set #1 | NEMI | NEMI | NEMI | Alouette | WJ-III |
| Set #2 | NEMI | NEMI | NEMI | Alouette | WJ-III |
| Set #3 | WASI | WASI | WASI | TOWRE | CMAT |
| Set #4 | WASI | WASI | WASI | TOWRE | WJ-III |
| Set #5 | WASI | WASI | WASI | TOWRE | CMAT |
| Set #4 | WASI | WASI | WASI | TOWRE | WJ-III |

*Note.* Alouette = Alouette reading test, CMAT = Comprehensive Mathematical Abilities Test, NEMI = Nouvelle Echelle Métrique de l’Intelligence-2,
WASI = Wechsler Abbreviated Scale of Intelligence, WJ-III = Woodcock-Johnson III Tests of Achievement.

**Supplementary Table 2.**
Descriptive statistics and correlations of behavioral variables for set #1 (n = 52; Girard et al., 2022)

| Variables | Cat. 1 | % | Cat. 2 | % | (1) | (2) | (3) | (4) | (5) | (6) | (7) |
| --- | --- | --- | --- | --- | --- | --- | --- | --- | --- | --- | --- |
| (1) Sex | male | 69.8 | female | 30.2 |  |  |  |  |  |  |  |
| (2) ADHD | no | 100.0 | yes | 0.0 | − |  |  |  |  |  |  |
|  | Mean | SD | Min | Max |  |  |  |  |  |  |  |
| (3) Age | 8.47 | .35 | 8.01 | 9.24 | -.03 | − |  |  |  |  |  |
| (4) IQ verbal | 4.83 | 1.42 | 2.00 | 7.00 | -.05 | − | -.20 |  |  |  |  |
| (5) IQ nonverb. | 4.49 | 1.35 | 2.00 | 7.00 | .19 | − | -.04 | **.46**** |  |  |  |
| (6) Arithmetic | 110.41 | 9.59 | 87.14 | 122.35 | .02 | − | .11 | **.27*** | **.39**** |  |  |
| (7) Vocabulary | 4.75 | 1.11 | 2.00 | 7.00 | -.00 | − | **-.44**** | **.51**** | **.29*** | **.28*** |  |
| (8) Reading | 105.92 | 8.37 | 86.25 | 119.77 | .08 | − | -.02 | .16 | .12 | **.34*** | **.32*** |

*Note.* Pearson’s correlation, * *p* ≤ .05,***p* ≤ .01. Cat. 1 = Category 1, Cat. 2 = Category 2. Significant (*p* < .05) correlations are bold.

**Supplementary Table 3.**
Descriptive statistics and correlations of behavioral variables for set #2 (n = 42; Schwarz et al., 2018)

| Variables | Cat. 1 | % | Cat. 2 | % | (1) | (2) | (3) | (4) | (5) | (6) | (7) |
| --- | --- | --- | --- | --- | --- | --- | --- | --- | --- | --- | --- |
| (1) Sex | male | 45.2 | female | 54.8 |  |  |  |  |  |  |  |
| (2) ADHD | no | 100.0 | yes | 0.0 | − |  |  |  |  |  |  |
|  | Mean | SD | Min | Max |  |  |  |  |  |  |  |
| (3) Age | 11.05 | 1.25 | 8.47 | 11.05 | -.18 | − |  |  |  |  |  |
| (4) IQ verbal | 4.81 | 1.40 | 2.00 | 7.00 | .12 | − | .22 |  |  |  |  |
| (5) IQ nonverb. | 3.93 | 1.52 | 1.00 | 7.00 | -.17 | − | -.20 | **.37*** |  |  |  |
| (6) Arithmetic | 98.07 | 25.13 | 60.00 | 150.00 | -.20 | − | **-.36*** | .21 | **.51**** |  |  |
| (7) Vocabulary | 4.36 | 1.36 | 1.00 | 7.00 | .06 | − | .02 | **.40**** | .27 | **.33*** |  |
| (8) Reading | 93.77 | 4.50 | 77.14 | 99.25 | .55 | − | .08 | .20 | .06 | **.31*** | .13 |

*Note.* Pearson’s correlation, * *p* ≤ .05,***p* ≤ .01. Cat. 1 = Category 1, Cat. 2 = Category 2. Significant (*p* < .05) correlations are bold.

**Supplementary Table 4.**
Descriptive statistics and correlations of behavioral variables for set #3 (n = 132; Suárez-Pellicioni et al., 2019)

| Variables | Cat. 1 | % | Cat. 2 | % | (1) | (2) | (3) | (4) | (5) | (6) | (7) | (8) |
| --- | --- | --- | --- | --- | --- | --- | --- | --- | --- | --- | --- | --- |
| (1) Sex | male | 47.0 | female | 53.0 |  |  |  |  |  |  |  |  |
| (2) ADHD | no | 81.8 | yes | 18.2 | -.11 |  |  |  |  |  |  |  |
|  | Mean | SD | Min | Max |  |  |  |  |  |  |  |  |
| (3) Age | 11.26 | 1.46 | 8.36 | 15.00 | **-.18*** | .05 |  |  |  |  |  |  |
| (4) IQ verbal | 55.67 | 9.57 | 33.00 | 78.00 | .06 | -.15 | -.16 |  |  |  |  |  |
| (5) IQ nonverb. | 53.66 | 9.56 | 29.00 | 72.00 | -.07 | -.12 | **-.20*** | **.35**** |  |  |  |  |
| (6) Arithmetic | 101.08 | 23.30 | 62.00 | 159.00 | -.02 | **-.20*** | -.14 | **.52**** | **.54**** |  |  |  |
| (7) Vocabulary | 53.57 | 9.97 | 34.00 | 80.00 | .03 | -.05 | **-.23**** | **.70**** | **.42**** | **.54**** |  |  |
| (8) Reading | 104.24 | 13.27 | 62.00 | 133.00 | .11 | -.12 | **-.20*** | **.43**** | **.33**** | **.48**** | **.47**** |  |
| (9) Phonological  Awareness | 98.55 | 15.84 | 59.00 | 133.00 | -.01 | -.12 | -.13 | **.47**** | **.47**** | **.45**** | **.58**** | **.54**** |

*Note.* Pearson’s correlation, * *p* ≤ .05,***p* ≤ .01. Cat. 1 = Category 1, Cat. 2 = Category 2. Significant (*p* < .05) correlations are bold.

**Supplementary Table 5.**
Descriptive statistics and correlations of behavioral variables for set #4 (n = 185; Lytle, McNorgan and Booth, 2019)

| Variables | Cat. 1 | % | Cat. 2 | % | (1) | (2) | (3) | (4) | (5) | (6) | (7) | (8) |
| --- | --- | --- | --- | --- | --- | --- | --- | --- | --- | --- | --- | --- |
| (1) Sex | male | 51.9 | female | 48.1 |  |  |  |  |  |  |  |  |
| (2) ADHD | no | 77.3 | yes | 22.7 | -.09 |  |  |  |  |  |  |  |
|  | Mean | SD | Min | Max |  |  |  |  |  |  |  |  |
| (3) Age | 10.48 | 1.62 | 7.50 | 14.38 | -.13 | -.01 |  |  |  |  |  |  |
| (4) IQ verbal | 56.68 | 9.84 | 33.00 | 80.00 | -.02 | .04 | -.08 |  |  |  |  |  |
| (5) IQ nonverb. | 54.00 | 8.81 | 29.00 | 72.00 | .08 | .04 | -.09 | **.45**** |  |  |  |  |
| (6) Arithmetic | 100.89 | 13.60 | 60.00 | 133.00 | .05 | **-.17*** | .00 | **.44**** | **.35**** |  |  |  |
| (7) Vocabulary | 54.70 | 10.39 | 33.00 | 79.00 | -.07 | .06 | -.08 | **.73**** | **.49**** | **.44**** |  |  |
| (8) Reading | 97.35 | 14.49 | 59.00 | 144.00 | **.16*** | **-.18*** | -.06 | **.39**** | **.34**** | **.45**** | **.39**** |  |
| (9) Phonological  Awareness | 98.23 | 13.76 | 67.00 | 127.00 | .09 | -.05 | -.06 | **.46**** | **.40**** | **.37**** | **.47**** | **.46**** |

*Note.* Pearson’s correlation, * *p* ≤ .05,***p* ≤ .01. Cat. 1 = Category 1, Cat. 2 = Category 2. Significant (*p* < .05) correlations are bold.

**Supplementary Table 6.**
Descriptive statistics and correlations of behavioral variables for set #5 (n = 56; Lytle, Prado & Booth, 2020)

| Variables | Cat. 1 | % | Cat. 2 | % | (1) | (2) | (3) | (4) | (5) | (6) | (7) | (8) |
| --- | --- | --- | --- | --- | --- | --- | --- | --- | --- | --- | --- | --- |
| (1) Sex | male | 42.9 | female | 57.1 |  |  |  |  |  |  |  |  |
| (2) ADHD | no | 83.9 | yes | 16.1 | -.21 |  |  |  |  |  |  |  |
|  | Mean | SD | Min | Max |  |  |  |  |  |  |  |  |
| (3) Age | 11.20 | 1.64 | 8.47 | 15.00 | -.23 | **.27*** |  |  |  |  |  |  |
| (4) IQ verbal | 59.04 | 8.97 | 37.00 | 76.00 | -.03 | -.18 | -.08 |  |  |  |  |  |
| (5) IQ nonverb. | 56.05 | 8.47 | 32.00 | 69.00 | -.15 | -.22 | -.23 | **.26^+^** |  |  |  |  |
| (6) Arithmetic | 111.45 | 22.79 | 62.00 | 159.00 | -.14 | -.22 | -.10 | **.47**** | **.53**** |  |  |  |
| (7) Vocabulary | 56.91 | 9.82 | 38.00 | 79.00 | .04 | -.04 | -.17 | **.73**** | **.31*** | **.38**** |  |  |
| (8) Reading | 109.75 | 11.95 | 86.00 | 133.00 | .01 | **-.36**** | -.23 | **.39**** | **.36**** | **.34*** | **.36**** |  |
| (9) Phonological  Awareness | 105.25 | 14.88 | 64.00 | 133.00 | -.09 | -.11 | -.17 | **.48**** | **.42**** | **.37**** | **.53**** | **.48**** |

*Note.* Pearson’s correlation, * *p* ≤ .05,***p* ≤ .01. Cat. 1 = Category 1, Cat. 2 = Category 2. Significant (*p* < .05) correlations are bold.

**Supplementary Table 7.**
Descriptive statistics and correlations of behavioral variables for set #6 (n = 68; Lytle, Hammer & Booth, 2020)

| Variables | Cat. 1 | % | Cat. 2 | % | (1) | (2) | (3) | (4) | (5) | (6) | (7) | (8) |
| --- | --- | --- | --- | --- | --- | --- | --- | --- | --- | --- | --- | --- |
| (1) Sex | male | 94.1 | female | 5.9 |  |  |  |  |  |  |  |  |
| (2) ADHD | no | 39.7 | yes | 60.3 | **-.31*** |  |  |  |  |  |  |  |
|  | Mean | SD | Min | Max |  |  |  |  |  |  |  |  |
| (3) Age | 10.34 | .94 | 8.59 | 11.96 | -.16 | -.06 |  |  |  |  |  |  |
| (4) IQ verbal | 58.43 | 10.56 | 22.00 | 77.00 | .22 | **-.24*** | -.03 |  |  |  |  |  |
| (5) IQ nonverb. | 55.47 | 9.67 | 22.00 | 72.00 | .22 | **-.28*** | -.09 | **.48**** |  |  |  |  |
| (6) Arithmetic | 104.47 | 13.07 | 72.00 | 134.00 | .19 | **-.28*** | .18 | **.44**** | **.57**** |  |  |  |
| (7) Vocabulary | 58.00 | 11.24 | 34.00 | 80.00 | **.40**** | **-.36**** | -.12 | **.75**** | **.65**** | **.51**** |  |  |
| (8) Reading | 102.75 | 16.63 | 58.00 | 140.00 | **.29*** | **-.30*** | -.07 | **.32**** | **.41**** | **.28*** | **.56**** |  |
| (9) Phonological  Awareness | 104.06 | 14.14 | 70.00 | 127.00 | **.26*** | -.23 | -.12 | **.57**** | **.60**** | **.38**** | **.66**** | **.52**** |

*Note.* Pearson’s correlation, * *p* ≤ .05,** *p* ≤ .01. Cat. 1 = Category 1, Cat. 2 = Category 2. Significant (*p* < .05) correlations are bold.

***Supplementary Table 8***

*Mixed-model analysis of GMV in the left IPS without covariates of language and cognitive measures.*

| **Fixed effects** | **Estimated**  **coefficient** | **SE** | **Lower** | **Upper** | ***df*** | ***t*** | ***p*** |
| --- | --- | --- | --- | --- | --- | --- | --- |
| (Intercept) | .443 | .00 | .446 | .450 | .53 | 120.14 | .05 |
| Arithmetic | -3.38e−4 | .00 | -.005 | .004 | 522.13 | -.14 | .89 |
| ADHD (1 – 0) | -.015 | .01 | -.027 | -.002 | 328.84 | **-2.26** | **.02** |
| Sex (2 – 1) | -.014 | .01 | -.027 | -3.99e−4 | 528.57 | -2.02 | .04 |
| Age | -.009 | .00 | -.011 | -.006 | 131.23 | **-5.90** | **< .001** |
| TIV | 2.06e-4 | 1.92e-5 | 1.68e-4 | 2.43e-4 | 392.76 | **10.69** | **< .001** |
| Sex*ADHD | -.020 | .01 | -.044 | .005 | 528.78 | -1.58 | .12 |
| **Random effects** | **Variance** | ***SD*** | ***ICC*** |  |  |  |  |
| ArithTest (Intercept) | .00 | .00 | .00 |  |  |  |  |
| Scanning_site (Intercept) | 4.99e-6 | .00 | .00 |  |  |  |  |
| Residual | .00 | .05 |  |  |  |  |  |

*Notes.* Random effects are correlated. *AIC* = -1606.08; *BIC* = -1487.18; R-squared marginal = .29; R-squared conditional = .29. Significant (*p* < .05) fixed effects are in bold.

***Supplementary Table 9***

*Mixed-model analysis of GMV in the right IPS without covariates of language and cognitive measures.*

| **Fixed effects** | **Estimated**  **coefficient** | **SE** | **Lower** | **Upper** | ***df*** | ***t*** | ***p*** |
| --- | --- | --- | --- | --- | --- | --- | --- |
| (Intercept) | .465 | .00 | .458 | .473 | .62 | 127.68 | .03 |
| Arithmetic | .003 | .00 | -9.21e−4 | .008 | 523.70 | 1.55 | .12 |
| ADHD (1 – 0) | -.005 | .01 | -.017 | .007 | 353.42 | -.84 | .40 |
| Sex (2 – 1) | -.003 | .01 | -.016 | .009 | 528.53 | -.55 | .58 |
| Age | -.007 | .00 | -.010 | -.005 | 152.42 | **-5.50** | **< .001** |
| TIV | 2.74e-4 | 1.79e-5 | 2.39e-4 | 3.09e-4 | 412.46 | **15.31** | **< .001** |
| Sex*ADHD | -.014 | .01 | -.036 | .009 | 528.73 | -1.19 | .23 |
| **Random effects** | **Variance** | ***SD*** | ***ICC*** |  |  |  |  |
| ArithTest (Intercept) | .00 | .00 | .00 |  |  |  |  |
| Scanning_site (Intercept) | 6.60e-6 | .00 | .00 |  |  |  |  |
| Residual | .00 | .05 |  |  |  |  |  |

*Notes.* Random effects are correlated. *AIC* = -1683.24; *BIC* = -1563.36; R-squared marginal = .40; R-squared conditional = .40. Significant (*p* < .05) fixed effects are in bold.

***Supplementary Table 10***

*Mixed-model analysis of GMV in the left AG without covariates of language and cognitive measures.*

| **Fixed effects** | **Estimated**  **coefficient** | **SE** | **Lower** | **Upper** | ***df*** | ***t*** | ***p*** |
| --- | --- | --- | --- | --- | --- | --- | --- |
| (Intercept) | .436 | .00 | .426 | .445 | .86 | 90.30 | .01 |
| Arithmetic | 3.88e-4 | .00 | -.003 | .004 | 528.01 | .24 | .81 |
| ADHD (1 – 0) | -.005 | .00 | -.013 | .004 | 486.52 | -1.05 | .29 |
| Sex (2 – 1) | .005 | .00 | -.003 | .014 | 468.04 | 1.21 | .23 |
| Age | -.006 | 9.99e-4 | -.008 | -.004 | 122.49 | **-5.56** | **< .001** |
| TIV | 3.16e-4 | 1.29e-5 | 2.90e-4 | 3.41e-4 | 520.35 | **24.37** | **< .001** |
| Sex*ADHD | .003 | .01 | -.013 | .020 | 527.62 | .41 | .68 |
| **Random effects** | **Variance** | ***SD*** | ***ICC*** |  |  |  |  |
| ArithTest (Intercept) | 1.88e-9 | 4.34e-5 | 1.48e-6 |  |  |  |  |
| Scanning_site (Intercept) | 3.35e-5 | .01 | .03 |  |  |  |  |
| Residual | .00 | .04 |  |  |  |  |  |

*Notes.* Random effects are correlated. *AIC* = -2037.90; *BIC* = -1915.06; R-squared marginal = .60; R-squared conditional = .61. Significant (*p* < .05) fixed effects are in bold.

***Supplementary Table 11***

*Mixed-model analysis of GMV in the left Hippocampus without covariates of language and cognitive measures.*

| **Fixed effects** | **Estimated**  **coefficient** | **SE** | **Lower** | **Upper** | ***df*** | ***t*** | ***p*** |
| --- | --- | --- | --- | --- | --- | --- | --- |
| (Intercept) | .449 | .00 | .442 | .455 | 1.13 | 132.09 | .00 |
| Arithmetic | -7.53e−4 | .00 | -.003 | .001 | 526.72 | -.67 | .51 |
| ADHD (1 – 0) | -.003 | .00 | -.009 | .003 | 395.60 | -1.14 | .26 |
| Sex (2 – 1) | -.002 | .00 | -.008 | .004 | 528.39 | -.63 | .53 |
| Age | .001 | 7.20e-4 | -2.45e−4 | .003 | 345.06 | 1.62 | .11 |
| TIV | 2.15e-4 | 9.10e-6 | 1.97e-4 | 2.33e-4 | 469.65 | **23.60** | **< .001** |
| Sex*ADHD | .004 | .01 | -.007 | .015 | 527.61 | .68 | .50 |
| **Random effects** | **Variance** | ***SD*** | ***ICC*** |  |  |  |  |
| ArithTest (Intercept) | 1.19e-5 | .00 | .02 |  |  |  |  |
| Scanning_site (Intercept) | 4.89e-6 | .00 | .01 |  |  |  |  |
| Residual | 6.32e-4 | .03 |  |  |  |  |  |

*Notes.* Random effects are correlated. *AIC* = -2410.71; *BIC* = -2282.73; R-squared marginal = .60; R-squared conditional = .61. Significant (*p* < .05) fixed effects are in bold.

***Supplementary Table 12***

*Mixed-model analysis of GMV in the right Hippocampus without covariates of language and cognitive measures.*

| **Fixed effects** | **Estimated**  **coefficient** | **SE** | **Lower** | **Upper** | ***df*** | ***t*** | ***p*** |
| --- | --- | --- | --- | --- | --- | --- | --- |
| (Intercept) | .413 | .00 | .408 | .417 | .57 | 190.94 | .03 |
| Arithmetic | -3.72e−4 | .00 | -.002 | .002 | 523.75 | -.36 | .72 |
| Age | .001 | 6.40e-4 | 2.41e-4 | .003 | 140.61 | **2.34** | **.02** |
| TIV | 2.05e-4 | 8.19e-6 | 1.89e-4 | 2.21e-4 | 405.45 | **25.02** | **< .001** |
| Sex (2 – 1) | .001 | .00 | -.005 | .007 | 522.14 | .38 | .70 |
| ADHD (1 – 0) | -2.85e−5 | .00 | -.005 | .005 | 304.26 | -.01 | .99 |
| Sex*ADHD | .010 | .01 | -1.60e−4 | .021 | 527.67 | 1.93 | .05 |
| **Random effects** | **Variance** | ***SD*** | ***ICC*** |  |  |  |  |
| ArithTest (Intercept) | 3.40e-6 | .00 | .01 |  |  |  |  |
| Scanning_site (Intercept) | 1.51e-6 | .00 | .00 |  |  |  |  |
| Residual | 5.16e-4 | .02 |  |  |  |  |  |

*Notes.* Random effects are correlated. *AIC* = -2521.25; *BIC* = -2390.77; R-squared marginal = .63; R-squared conditional = .63. Significant (*p* < .05) fixed effects are in bold.


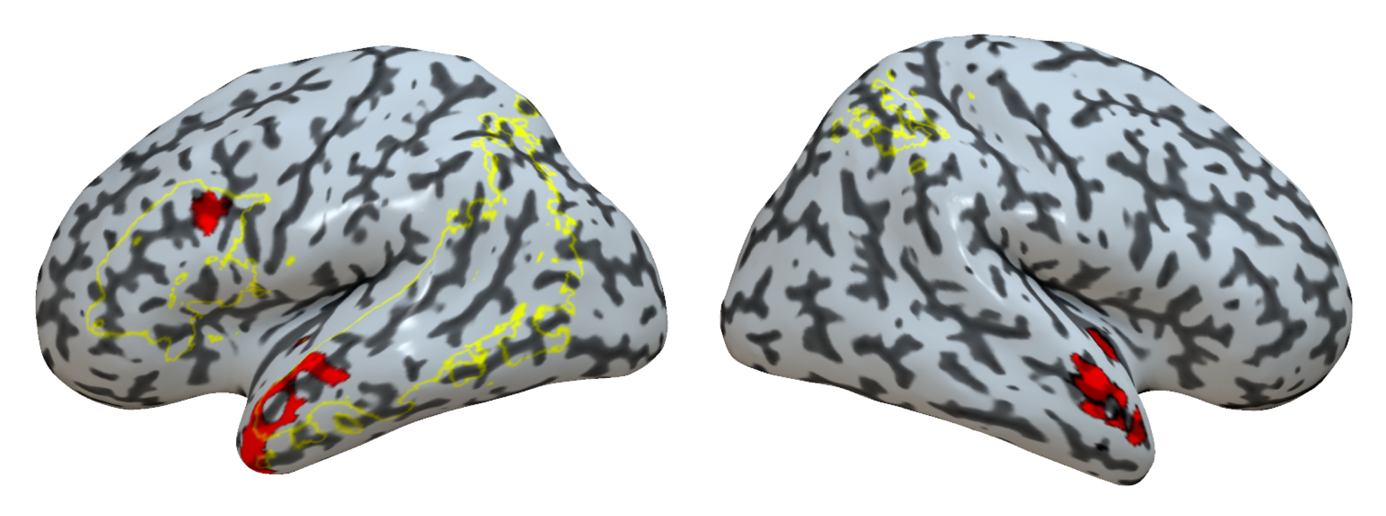


**Supplementary Figure S1.** Whole-brain regression analysis. In red are represented the brain regions in which a relation between arithmetic score and GMV was found. Yellow outlines delineate the border of a mask representing the union of all ROIs used in the main analysis. The analysis showed a relation between arithmetic score and GMV in the left IFG (MNI coordinates: x = -36, y = 14, z =26, Z = 5.42, pFWE < .001, volume = .57 cc), the left MTG (MNI coordinates: x = -57, y = 9, z =-24, Z = 6.08, pFWE < .001, volume = 3.12 cc), and the right MTG (MNI coordinates: x = 44, y = -9, z =-16, Z = 5.99, pFWE < .001, volume = 2.32 cc).
