# Supplementary material for: Arithmetic skills are associated with left fronto-temporal gray matter volume in 536 children and adolescents
Source: NPJ Sci Learn. 2023 Dec 8;8:56. doi: 10.1038/s41539-023-00201-x (PMC10709444; doi:10.1038/s41539-023-00201-x)
Supplement: Supplementary file 2 — Reporting Summary [file 41539_2023_201_MOESM2_ESM.pdf]

nature portfolio

Corresponding author(s): Dr. Nurit Viesel-Nordmeyer

Last updated by author(s): YYYY-MM-DD

Reporting Summary

Nature Portfolio wishes to improve the reproducibility of the work that we publish. This form provides structure for consistency and transparency in reporting. For further information on Nature Portfolio policies, see our Editorial Policies and the Editorial Policy Checklist.

Statistics

For all statistical analyses, confirm that the following items are present in the figure legend, table legend, main text, or Methods section.

n/a	Confirmed	
☐	☒	The exact sample size (*n*) for each experimental group/condition, given as a discrete number and unit of measurement
☐	☒	A statement on whether measurements were taken from distinct samples or whether the same sample was measured repeatedly
☐	☒	The statistical test(s) used AND whether they are one- or two-sided<br>*Only common tests should be described solely by name; describe more complex techniques in the Methods section.*
☐	☒	A description of all covariates tested
☐	☒	A description of any assumptions or corrections, such as tests of normality and adjustment for multiple comparisons
☐	☒	A full description of the statistical parameters including central tendency (e.g. means) or other basic estimates (e.g. regression coefficient) AND variation (e.g. standard deviation) or associated estimates of uncertainty (e.g. confidence intervals)
☐	☒	For null hypothesis testing, the test statistic (e.g. *F*, *t*, *r*) with confidence intervals, effect sizes, degrees of freedom and *P* value noted<br>*Give P values as exact values whenever suitable.*
☒	☐	For Bayesian analysis, information on the choice of priors and Markov chain Monte Carlo settings
☒	☐	For hierarchical and complex designs, identification of the appropriate level for tests and full reporting of outcomes
☒	☐	Estimates of effect sizes (e.g. Cohen's *d*, Pearson's *r*), indicating how they were calculated

Our web collection on statistics for biologists contains articles on many of the points above.

Software and code

Policy information about availability of computer code

Data collection	MRI data were collected with a Siemens Prisma 3T MRI scanner (Siemens Healthcare, Erlangen, Germany) at the CERMEP Imagerie du vivant in Lyon, France as well as Siemens 3 T Trio-Tim Scanner (Siemens Healthcare, Erlangen, Germany) at the CAMRI=Northwestern University Center for Advanced Magnetic Resonance Imaging at the greater Chicago metropolitan area in the United States (US)
Data analysis	Data were analyzed using the Computational Anatomy Toolbox (Cat 12)61 within the Statistical Parametric Mapping Software Package (SPM 12)

For manuscripts utilizing custom algorithms or software that are central to the research but not yet described in published literature, software must be made available to editors and reviewers. We strongly encourage code deposition in a community repository (e.g. GitHub). See the Nature Portfolio guidelines for submitting code & software for further information.

Data

Policy information about availability of data

All manuscripts must include a data availability statement. This statement should provide the following information, where applicable:

- Accession codes, unique identifiers, or web links for publicly available datasets
- A description of any restrictions on data availability
- For clinical datasets or third party data, please ensure that the statement adheres to our policy

Raw MRI data are available on Open Neuro for sets #3 (https://openneuro.org/datasets/ds001486/versions/1.3.1), #4 (https://openneuro.org/datasets/ds001894/

versions/1.4.1), #5 (https://openneuro.org/datasets/ds002886/versions/1.1.0), and #6 (https://openneuro.org/datasets/ds002424/versions/1.2.0). Individual behavioral data and GMV maps for all datasets are available from Zenodo, as well as whole-brain unthresholded t-maps corresponding to Fig. 1 and Fig. 2 (https://doi.org/10.5281/zenodo.6993462).

Human research participants

Policy information about studies involving human research participants and Sex and Gender in Research.

Reporting on sex and gender	Findings just comply to sex, not to gender. Sex was considered in the studies design (female = 43.7 %) and determinend based on reporting of particpants and/or adults. Data are shared without retraceable data information including sex. In the analyses, sex was considered as control variable.
Population characteristics	The final sample consisted in 536 french-speaking (n = 95) or US-american-speaking (n = 441) children and adolescents from age 7.5 to 15 (mean = 10.58). SES ranged from relatively low to relatively high. Age was considered as covariate in the ANCOVA analyses as well as age*skills interaction were controlled. IQ was also in the normal to superior range. There are no treatment categories to report because of using MRI data.
Recruitment	Participants were recruited using print (public transit, magazine) and electronic (google, Facebook) advertisement, community events, and brochures sent to schools. Exclusion criteria included hearing deficit, MRI contraindication, history of neurological and psychiatric disorders, prematurity less than 36 weeks, and medication affecting central nervous system processing. Children with a diagnosis of ADHD were excluded from all datasets except set #6. Children with ADHD who were included in that dataset, however, were instructed to not take stimulant medication for ADHD for at least 24 hours prior to the testing sessions
Ethics oversight	Data collection for set #1 and set #2 was approved by a French national ethics committee (CPP Lyon Sud-Est II), while data collection for set #3 to set #6 was approved by the Institutional Review Board at Northwestern University in the US.

Note that full information on the approval of the study protocol must also be provided in the manuscript.

Field-specific reporting

Please select the one below that is the best fit for your research. If you are not sure, read the appropriate sections before making your selection.

☒ Life sciences ☐ Behavioural & social sciences ☐ Ecological, evolutionary & environmental sciences

For a reference copy of the document with all sections, see nature.com/documents/nr-reporting-summary-flat.pdf

Life sciences study design

All studies must disclose on these points even when the disclosure is negative.

Sample size	Sample size is determined because of the available access of data for secondary data analysis.
Data exclusions	Three participants had to be excluded from the present analyses because of missing behavioral data of interest. One participant had to be excluded because of image artifacts.
Replication	*Describe the measures taken to verify the reproducibility of the experimental findings. If all attempts at replication were successful, confirm this OR if there are any findings that were not replicated or cannot be reproduced, note this and describe why.*
Randomization	*Describe how samples/organisms/participants were allocated into experimental groups. If allocation was not random, describe how covariates were controlled OR if this is not relevant to your study, explain why.*
Blinding	*Describe whether the investigators were blinded to group allocation during data collection and/or analysis. If blinding was not possible, describe why OR explain why blinding was not relevant to your study.*

Reporting for specific materials, systems and methods

We require information from authors about some types of materials, experimental systems and methods used in many studies. Here, indicate whether each material, system or method listed is relevant to your study. If you are not sure if a list item applies to your research, read the appropriate section before selecting a response.

Materials & experimental systems

n/a	Involved in the study
☒	☐ Antibodies
☒	☐ Eukaryotic cell lines
☒	☐ Palaeontology and archaeology
☒	☐ Animals and other organisms
☒	☐ Clinical data
☒	☐ Dual use research of concern

Methods

n/a	Involved in the study
☒	☐ ChIP-seq
☒	☐ Flow cytometry
☐	☒ MRI-based neuroimaging

Magnetic resonance imaging

Experimental design

Design type: *Indicate task or resting state; event-related or block design.*

Design specifications: *Specify the number of blocks, trials or experimental units per session and/or subject, and specify the length of each trial or block (if trials are blocked) and interval between trials.*

Behavioral performance measures: *State number and/or type of variables recorded (e.g. correct button press, response time) and what statistics were used to establish that the subjects were performing the task as expected (e.g. mean, range, and/or standard deviation across subjects).*

Acquisition

Imaging type(s): T1 structural

Field strength: 3 Tesla

Sequence & imaging parameters: Parameters of the anatomical scan for set #1 and #2 were as follow: TR=3500 ms, TE=2.24 ms, flip angle=8°, matrix size=256 × 256, slice thickness=0.90 mm, number of slices=192, voxel size resolution = 0.875 mm isotropic. In sets #3 to #6, brain imaging data were acquired using either a 16-channel or a 32-channel head coil and a Siemens 3 T Trio-Tim Scanner (Siemens Healthcare, Erlangen, Germany). Parameters of the anatomical scan for sets #3 to #6 were as follow: TR=2300 ms, TE = 3.36 ms, flip angle=9 °, matrix size=256 ×256, slice thickness=1 mm, number of slices=160, voxel size resolution=1 mm isotropic.

Area of acquisition: whole-brain

Diffusion MRI ☐ Used ☒ Not used

Preprocessing

Preprocessing software: the Computational Anatomy Toolbox (Cat 12)61 within the Statistical Parametric Mapping Software Package (SPM 12)

Normalization: images were spatially normalized using DARTEL registration with an MNI template also provided by the CAT12 toolbox

Normalization template: MNI

Noise and artifact removal: We used cat12 to identify volumes with motion artifacts.

Volume censoring: *Define your software and/or method and criteria for volume censoring, and state the extent of such censoring.*

Statistical modeling & inference

Model type and settings: Whole-brain voxel-based regression analyses were conducted on GMV images. ANCOVAs with whole-brain GMV as factor and several covariates were computed to detect brain regions in which GMV was associated with individual differences in arithmetic skills

Effect(s) tested: In the main analysis of the full sample (n=536), the variable of interest was arithmetic skill while covariates were measures of vocabulary, reading, verbal and non-verbal IQ, age, sex, ADHD status, type of scanner and TIV. First order interactions between age and each skill was added. Follow up regression analyses were also conducted with the youngest children and the oldest children, based on a median split of age. These included the same covariates as mentioned before, with the exception of the interactions with age.

Specify type of analysis: ☒ Whole brain ☐ ROI-based ☐ Both

Statistic type for inference (See Eklund et al. 2016): cluster-level correction

Correction	All statistical maps were thresholded using a voxel-level threshold of $p \le .001$ and a cluster-level threshold of $p \le .05$, family-wise error corrected for multiple comparisonswhole-brain statistical map to assess brain activations

Models & analysis

n/a	Involved in the study
☒	☐ Functional and/or effective connectivity
☒	☐ Graph analysis
☒	☐ Multivariate modeling or predictive analysis
